# The General Medical Council

**Published:** 1905-12-09

**Authors:** 


### The General Medical Council.

The eighty-second Session of the General Medical Council, which commenced on the 28th ultimo, has not been an eventful one; and, indeed, cannot be said to have produced any discussion of grave professional importance. The late President, Sir William Turner, who remained a member of the Council during the first session after his retirement from the Presidency, has now resigned his seat; and, for the first time for thirty-two years, the Council is deprived of his ripe wisdom and of his great experience. He is replaced, as representative of the University of Edinburgh, by Sir Thomas Fraser, Professor of Materia Medica and Clinical Medicine in the University; and the Council is increased in number by the representative of the new University of Sheffield, Dr. Cocking. There is a manifest fairness in admitting the representatives of newly created licensing bodies; although it may be questioned whether such bodies are not already too numerous, and whether the Council itself has not become too large for the prompt and convenient despatch of business.

Perhaps the most interesting announcement contained in the President's address at the opening of the session was to the effect that an application had been made to His Majesty on behalf of the Japanese Government, requesting that steps may be taken to recognise Japan as one of the countries to which the Medical Act of 1886 applies, and thus to enable Japanese medical practitioners to practise medicine in the Straits Settlements as well as in other parts of the British Empire. In consequence of this application, it has devolved upon the Privy Council to ascertain whether duly qualified British subjects are permitted to practise in Japan; and, if they are, it will be the duty of the General Medical Council to inquire into the conditions of medical education and licensing in that kingdom, and to consider what diplomas granted there shall be recognised for registration on the foreign list in this country. If any conclusions may be drawn from the reports of Japanese medicine and surgery in Manchuria, there can be little doubt of the complete eligibility of Japanese graduates for the privileges which they seek.

The efforts of Sir John Batty Tuke and other members of Parliament to obtain the passage of an enactment to check the evasion of the Medical and Dental Acts by unqualified persons formed into joint stock companies for the purpose have so far not been successful; and, in view of the political turmoil incidental to a change of ministry and to the election of a new House of Commons, it would probably be over sanguine to expect any effective legislation for this purpose in the near future. It is the more satisfactory to find that the Council itself has been able to take some steps for the protection of its constituents, and that on the first instant it adjudicated upon the case of seven persons, all of them registered as dentists who were in practice before July 22, 1878, and who had signed the articles of association of a limited company formed for the purpose of transferring a dentist's business to G. Guy White and F. Sinclair Kennedy, unqualified and unregistered persons, who, on May 2, 1904, were convicted at the Marlborough Street Police Court for infringement of the Dentists Act by carrying on business at 215 Piccadilly under the title of dentists and surgeon-dentists. Alleged infringements of the Dentists Act are not heard by the Council as a whole, but by its Dental Committee, which reports the ascertained facts to the Council as matters upon which to adjudicate. The report presented in this case showed that some of the accused persons had only signed the articles, while others had taken an active part in the transaction, and had been concerned in the issue of advertisements of an objectionable character. The ultimate decision of the Council was that the names of Algernon Frederick Green and of George Blount should be erased from the dentists' register, and that the further consideration of the cases of the other five defendants should be postponed until the next session of the Council, when they will be permitted to adduce evidence of having abandoned the practices complained of. The proof thus given that the Council has a longer arm than had been supposed cannot fail to be advantageous, and to check improper combinations between qualified and unqualified persons.

